# Sleep Duration, Schedule and Quality among Urban Chinese Children and Adolescents: Associations with Routine After-School Activities

**DOI:** 10.1371/journal.pone.0115326

**Published:** 2015-01-22

**Authors:** Xiaoxiao Jiang, Louise L. Hardy, Louise A. Baur, Ding Ding, Ling Wang, Huijing Shi

**Affiliations:** 1 School of Public Health and Key Laboratory of Public Health Safety, Fudan University, Shanghai, China; 2 Prevention Research Collaboration, School of Public Health, University of Sydney, Sydney, Australia; Osaka University, JAPAN

## Abstract

**Background:**

With rapid urbanization accompanied by lifestyle changes, children and adolescents living in metropolitan areas are faced with many time use choices that compete with sleep. This study reports on the sleep hygiene of urban Chinese school students, and investigates the relationship between habitual after-school activities and sleep duration, schedule and quality on a regular school day.

**Methods:**

Cross-sectional, school-based survey of school children (Grades 4–8) living in Shanghai, China, conducted in 2011. Self-reported data were collected on students’ sleep duration and timing, sleep quality, habitual after-school activities (i.e. homework, leisure-time physical activity, recreational screen time and school commuting time), and potential correlates.

**Results:**

Mean sleep duration of this sample (mean age: 11.5-years; 48.6% girls) was 9 hours. Nearly 30% of students reported daytime tiredness. On school nights, girls slept less (*p*<0.001) and went to bed later (*p*<0.001), a sex difference that was more pronounced in older students. Age by sex interactions were observed for both sleep duration (*p*=0.005) and bedtime (*p*=0.002). Prolonged time spent on homework and mobile phone playing was related to shorter sleep duration and later bedtime. Adjusting for all other factors, with each additional hour of mobile phone playing, the odds of daytime tiredness and having difficulty maintaining sleep increased by 30% and 27% among secondary students, respectively.

**Conclusion:**

There are sex differences in sleep duration, schedule and quality. Habitual activities had small but significant associations with sleep hygiene outcomes especially among secondary school students. Intervention strategies such as limiting children’s use of electronic screen devices after school are implicated.

## Introduction

Adequate sleep plays a crucial role in the physical, mental and cognitive development of children and adolescents. [1–3] Insufficient sleep is linked to impaired mental health [4] and school performance, [5] obesity, [6] and health risk behaviors. [7] The sleep duration of children and adolescents has declined by nearly one hour per night during the past century, especially in countries in Asia and North America. [8] Among the Chinese pediatric population, short sleep duration and sleep problems including sleep onset latency (i.e. difficulty initiating sleep), sleep disturbance (i.e. difficulty maintaining sleep), and daytime tiredness or sleepiness are prevalent, [9–10] with sleep duration being less than for American children [11]. Although there is no current international consensus on optimal sleep duration for each age group, [12] many factors, such as age, health status and the urban context, contribute to children’s sleep behavior and quality. [13–16]

Sociocultural [17] and family environments [18] have been explored to explain Chinese children’s sleep behaviors, but few studies have focused on the relationship between children’s and adolescents’ routine activities outside school hours and sleep outcomes. It is possible that children today, consciously or not, trade their sleep time for other activities that are pressing or of greater interest. In Asian children, academic performance and pressure are strongly associated with sleep duration, especially among adolescents. [19–20] However, there has been little investigation of the actual time that children spend studying after school, which may have an independent association with sleep apart from academic pressure. Among nighttime activities, inverse associations between electronic screen entertainment (i.e. TV viewing, playing computers and videogames) and sleep have been observed in both developed and developing countries [21–23] though the evidence was inconsistent regarding mobile phone use. [24] Western studies [25–26] have emphasized the benefits of delaying school start times, however, in China long distances travelled between home and school and traffic congestion may compromise such efforts. Furthermore, most Chinese studies have focused on younger children and there are no studies in adolescents.

Rapid urbanization, accompanied by tremendous changes in lifestyle, is affecting young people in Shanghai and other developed areas of China. Children and adolescents are faced with a new range of time use choices and prolonged commuting times that compete with sleep time and possibly interfere with sleep, schedule and quality. Therefore, in this study of older children and adolescents living in the Shanghai inner city area, we aimed to: 1) report the sleep duration, schedule, and sleep quality and 2) examine the associations between time spent on routine activities outside school hours and a range of sleep hygiene outcomes.

## Materials and Methods

### Sampling and procedures

A cross-sectional, school-based questionnaire survey was conducted among Shanghai inner city students from Grades 4 to 8 (both elementary and secondary grades) between October and November 2011, a period when there were no public holidays or exams. Trained field staff administered a questionnaire to the students during a school visit. Parents or guardians filled out separate questionnaires which were submitted by the student within a week after the first submission. Informed consent was well-explained for all students and their parents, and signed by both students themselves and their parents or guardians voluntarily before participating in the study. The study was approved by the Ethics Review Board of Fudan University (IRB # 2011–12–0321)”.

A multi-stage random cluster sampling design was used to select districts, schools and classes. The first stage involved the random selection of four districts in inner city areas of Shanghai. The second stage involved the random selection of schools (three elementary schools and three middle schools from each district) and the third stage involved the random selection of classes stratified by grade (213 classes from 24 schools). Private or international schools in Shanghai were not included in this study because of the difference in school curricula.

### Measures

Students’ sex, date of birth, weight and height were extracted from the Shanghai annual physical examination database. [27] Weight (kg) and height (m) were measured by authorized clinical staff in the spring of 2011 and body mass index (BMI) was calculated. BMI was classified based on the International Obesity Taskforce (IOTF) age-sex specific BMI cut-points for the Asian pediatric population. [28] A questionnaire was given to parents to provide their educational level (whether or not they had a university degree) and the number of children at home.

**Sleep hygiene (timing, duration, and quality).** Students were asked to report what time they usually go to bed and wake up on a regular school day (timing). Sleep duration was calculated as the time lapse between bedtime and wake up time.

Sleep quality was assessed using the Multidimensional Sub-health Questionnaire of Adolescents (MSQA), which shows good internal reliability and criterion validity among Chinese adolescents. [29] Students reported “For the past six months, how many weeks/months in total have you been: (1) having difficulty falling asleep?; (2) having difficulty staying asleep at night?; (3) feeling tired and lacking energy during the day?” to indicate three sleep problems as ‘Had difficulty initiating sleep’, ‘Had difficulty maintaining sleep’, and ‘Daytime tiredness’, respectively. For each question, response categories were none; less than one week; 1–2 weeks; more than 2 weeks; more than 1 month; more than 2 months; and, more than 3 months. Responses were dichotomized as not having sleep problems (none and less than one week) or having sleep problems (remaining responses).

**Recreational screen time and mobile phone playing.** Students reported the duration of weekday after-school screen-time (i.e. watching TV, playing on a computer (desktop, laptop or tablet devices) and playing videogames), which were summed and categorized according to recommendations of <2 hours or ≥2 hours. [30] The duration of mobile phone playing was determined by the question ‘How many hours and minutes have you spent playing the mobile phone (i.e. texting, playing games, or surfing on internet) after school?’, and the 10-day test-retest reliability of the questions was satisfactory (intraclass correlation coefficient = 0.78 and 0.74 for screen-time and time spent mobile phone playing, respectively).

**Study load and academic pressure.** For the study load, students reported time they spent on homework on an average school day, as measured by the question “How many hours and minutes do you usually spend on doing your homework with or without a computer on an average school night?” (Test-retest reliability of intraclass correlation coefficient = 0.85 for total time spent on homework). For self-perceived academic pressure, students also responded to the question “How much are you stressed about your academic performance?” with a five-point Likert scale ranging from “very much” to “not at all”.

**Commuting time.** Students reported the time spent commuting to and from home to school by car, public transport, cycling or walking, separately. These items were summed to calculate total commuting time.

**Leisure-time physical activity.** After-school leisure-time physical activity (LTPA) was measured by asking students how long they spent in light (e.g. walking), moderate (e.g. jogging, badminton) and vigorous (e.g. tennis, swimming) physical activity and summed to determine total LTPA.

### Statistical analysis

Main outcome variables were sleep duration, sleep schedule (represented by time going to bed, or bedtime), and sleep quality (represented by having or not having sleep problems). One-way ANOVA and the Chi-square test were used to describe the sleep duration, timing, and the prevalence of sleep problems across age groups respectively. Age and sex interactions for sleep duration and bedtime were tested by two-way ANOVA.

Multiple linear regression analysis was used to examine the potential correlates of sleep duration and bedtime (transformed into decimals). Separate analyses were conducted for school levels (i.e. elementary and secondary), as different patterns of sleep are expected at different developmental stages. Multivariate logistic regression was used for the three sleep quality outcomes. Multicollinearity diagnostic statistics were computed and the VIF values ranged from 1.01 to 1.12 (i.e., no evidence of multicollinearity). For multivariate analysis, the list-wise deletion method was used for handling missing data and the students were excluded from analyses if any single value was missing. The difference in demographic characteristics between the excluded students and the remaining students was compared by t-test or Chi-square test. Compared with students with complete data, more boys than girls (41.8% vs 33.7%, p<0.001) were with missing information of any of the independent variable. Due to missing data, the proportions of participants excluded from the analysis for elementary and secondary students were 34.7% and 42.9%, respectively. Sensitivity analyses were conducted by re-analyzing data including missing values on covariates as a separate category. Furthermore, to examine potential effect modification by parental education, we tested the interaction terms between parental education and each independent variable. Data were analyzed in 2013, using SPSS (version 21 for Windows, Chicago, IL). P values were two sided with a significance level of 0.05.

## Results

A total of 6,247 (response rate: 94.8%) students with parental data completed the survey. Table 1 summarizes the characteristics of the sample. The mean age was 11.5 years (range: 8.08–16.71 years), 48.6% were girls and 41.7% were in elementary school. The numbers of participants in each age group and school grade were distributed evenly.

**Table 1:** Sample characteristics.

	**Total Sample (n = 6247)**
	**n (%)**
Sex (girls)	3034 (48.6)
Age (years old) group	
<10	1291 (20.7)
10–11	1167 (18.7)
11–12	1351 (21.6)
12–13	1146 (18.3)
>13	1292 (20.7)
School grade	
4	1279 (20.5)
5	1326 (21.2)
6	1310 (21.0)
7	1169 (18.7)
8	1163 (18.6)
Father with university degree	1666 (27.4)
Mother with university degree	1339 (22.0)
Lives with a sibling	1513 (25.0)
Self-reported academic pressure	
Not at all or seldom	958 (16.7)
A little	2738 (47.8)
Yes or very much	2036 (35.5)
Daily screen time ≥ 2 hours	686 (12.4)
Overweight/obese ^a^	2382 (40.1)

^a^ According to International Task Force on Obesity Asian reference, sex-age-specific BMI cut-offs that correspond to overweight BMI of 23kg/m^2^ at age 18 years.

Sleep duration, sleep timing and the prevalence of sleep problems by age group and sex are shown in Table 2. The mean sleep duration on a regular school night was 9 hours, with the average bedtime approximately 9:30 pm and wake up time around 6:30 am. Older children woke up earlier, went to bed later and had a shorter sleep duration than younger ones (*p* for trend <0.001). Overall, 19% of the students reported having difficulty initiating sleep, 15.6% reported having difficulty maintaining sleep and almost one third (29.7%) reported daytime tiredness. There were significant differences among age groups for difficulty maintaining sleep (*p*=0.004) and daytime tiredness (*p*<0.001), but not for difficulty initiating sleep (*p*=0.371).

**Table 2:** Sleep duration, sleep timing and sleep problems by age group and sex (N = 6274).

	**Age group**						**Sex**		
	**<10 years**	**10–11 years**	**11–12 years**	**12–13 years**	**≥13 years**	***p***	**Boys**	**Girls**	***p***
**Sleep duration (hours)**	9.58(0.67)	9.43(0.70)	8.93(0.78)	8.67(0.71)	8.28(0.84)	<0.001	9.03(0.89)	8.92(0.88)	<0.001
**Sleep schedule ^a^**									
Bedtime (pm)	9.07(0.63)	9.18(0.66)	9.47(0.66)	9.67(0.66)	10.00(0.76)	<0.001	9.44(0.75)	9.52(0.76)	<0.001
Wake up time (am)	6.67(0.43)	6.63(0.43)	6.40(0.39)	6.34(0.39)	6.29(0.43)	<0.001	6.48(0.45)	6.45(0.43)	0.023
**Sleep problems (%)**									
Had difficulty initiating sleep	20.8	18.5	17.8	18.3	19.4	0.37	19.8	18.1	0.10
Had difficulty maintaining sleep	15.0	14.8	13.4	16.4	18.7	0.004	15.5	15.8	0.80
Daytime tiredness	25.2	25.7	28.4	31.3	37.8	<0.001	27.2	32.3	<0.001

^a^ Minutes were divided by 60 and multiplied by 100 and added to number of hours to generate a metric variable

Girls went to bed later, woke up earlier and slept less than boys (Table 2). Age by sex interactions were found for both sleep duration (*p*=0.005) and bedtime (*p*=0.002). Sex differences increased with age. For sleep problems, the only sex difference detected was among secondary school students, with more secondary school girl students reporting difficulty in maintaining sleep (*p*<0.001) and daytime tiredness (*p*<0.001).

Table 3 shows variables associated with sleep duration. For all students, sleep duration was inversely associated with time spent on homework, commuting and mobile phone playing, adjusting for potential confounders. The largest effect size was for homework, especially for secondary students. For secondary school boys, <2 hours daily screen-time was associated with longer sleep duration, while among secondary school girls, participating in less LTPA after school was associated with shorter sleep duration.

**Table 3:** Associations (indicated by regression coefficients) of weekday habitual after-school activity factors and sleep duration by school level and sex.

**School level**	**Elementary school (Grade 4 and 5)**		**Secondary school (Grade 6,7 and 8)**	
	**Beta**	***P***	**Beta**	***P***
**Boys**				
Commuting time (hour)	-0.097	**0.004**	-0.088	**0.003**
After-school LTPA (hour)	0.029	0.40	0.026	0.38
Homework time (hour)	-0.124	**<0.001**	-0.184	**<0.001**
Screen time (0= < 2 hours/ day, 1 = ≥ 2 hours/ day)	-0.016	0.65	-0.075	**0.013**
Playing mobile phone (hour)	-0.081	**0.024**	-0.119	**<0.001**
**Girls**				
Commuting time (hour)	-0.081	**0.017**	-0.079	**0.003**
After-school LTPA (hour)	-0.025	0.47	0.059	**0.030**
Homework time (hour)	-0.095	**0.005**	-0.294	**<0.001**
Screen time (0= < 2 hours/ day, 1 = ≥ 2 hours/ day)	-0.034	0.34	0.018	0.53
Playing mobile phone (hour)	-0.100	**0.004**	-0.092	**0.001**

All models were adjusted for age, BMI categories, parents’ education, whether or not lived with a sibling and academic pressure.

For the variables associated with school night bedtime (Table 4), more time spent on home work was found to be associated with later bedtime in all students. With the exception of elementary school boys, mobile phone playing was related to a later bedtime, especially in secondary school students. Secondary school girls who participated in less LTPA after school and ≥ 2 hours/day of screen-time had a later bedtime. School commuting time was not associated with bedtime in any of the models.

**Table 4:** Associations (indicated by regression coefficients) of weekday habitual after-school activity factors and bedtime by school level and sex.

**Age group**	**Elementary school (Grade 4 and 5)**		**Secondary school (Grade 6,7 and 8)**	
	**Beta**	***P***	**Beta**	***P***
**Boys**				
Commuting time (hour)	0.012	0.72	-0.019	0.51
After-school LTPA (hour)	-0.043	0.20	-0.043	0.14
Homework time (hour)	0.109	**0.001**	0.219	**<0.001**
Academic pressure (0 = not at all, 4 = very much, 0–4)	0.082	**0.015**	0.137	**<0.001**
Screen time (0= < 2 hours/ day, 1 = ≥ 2 hours/ day)	0.045	0.20	-0.016	0.58
Playing mobile phone (hour)	-0.049	0.17	0.121	**<0.001**
**Girls**				
Commuting time (hour)	0.042	0.21	-0.002	0.94
After-school LTPA (hour)	-0.012	0.72	-0.067	**0.013**
Homework time (hour)	0.078	**0.022**	0.303	**<0.001**
Screen time (0= < 2 hours/ day, 1 = ≥ 2 hours/ day)	0.077	**0.027**	0.007	0.81
Playing mobile phone (hour)	0.070	**0.046**	0.079	**0.005**

All models were adjusted for age, BMI categories, parents’ education, whether or not lived with a sibling and academic pressure.

Table 5 presents correlates of sleep problems. In elementary school, girls were less likely to have difficulty initiating (AOR[95%CI] = 0.73[0.57–0.94]) or maintaining sleep (AOR[95%CI] = 0.65[0.49–0.85]) than boys, while in secondary school, girls were more likely to report daytime tiredness (AOR[95%CI] = 1.35[1.11–1.64]), after adjusting for other factors. Homework time was independently associated with difficulty maintaining sleep and daytime tiredness among only secondary school students. Similarly, the adjusted odds of difficulty maintaining sleep and daytime tiredness increased by 27% and 37%, respectively, among secondary school students for each additional hour of mobile phone playing. We didn’t not find evidence for interactions between parental education and other independent variables (all p>0.05). As a sensitivity analysis, we reran all models with missing values of covariates coded as a category, which reduced case exclusion to 15.2% and 24.6% for elementary and secondary students. Results from sensitivity analysis were consistent with our original analysis, indicating robustness in our findings.

**Table 5:** Associations (indicated by odd ratios) of weekday habitual after-school activity factors and sleep quality problems by school level.

** **	**Elementary school (Grade 4 and 5)**					
	**Difficulty initiating sleep**		**Difficulty maintaining sleep**		**Day time tiredness**	
** **	**OR (95% CI)**	**AOR(95% CI)^a^**	**OR (95% CI)**	**AOR(95% CI)^a^**	**OR (95% CI)**	**AOR(95% CI)^a^**
Being a girl	**0.76(0.63–0.93)**	**0.73(0.57–0.94)**	**0.80(0.64–0.99)**	**0.65(0.49–0.85)**	1.06(0.89–1.28)	1.06(0.84–1.33)
After-school LTPA (hour)	1.00 (1.00–1.00)	1.00 (1.00–1.00)	1.00 (1.00–1.00)	1.00(1.00–1.00)	1.00 (1.00–1.00)	1.00 (1.00–1.00)
Commuting time (hour)	1.00(1.00–1.00)	1.00(1.00–1.00)	1.00(0.99–1.00)	1.00(0.99–1.00)	0.99(0.99–1.00)	1.00(1.00–1.00)
Homework time (hour)	0.98(0.90–1.07)	0.98(0.88–1.08)	1.08(0.98–1.18)	0.98(0.87–1.09)	**1.10(1.02–1.18)**	1.07(0.97–1.17)
Screen time >= 2 hours	0.85(0.62–1.17)	0.78(0.51–1.20)	1.20(0.87–1.67)	1.34(0.88–2.05)	1.03(0.78–1.36)	0.98(0.67–1.43)
Playing mobile phone (hour)	**1.32(1.01–1.72)**	1.43(0.98–2.07)	1.32(1.00–1.76)	1.19(0.79–1.77)	1.29(0.99–1.67)	1.34(0.94–1.93)
	**Secondary school (Grade 6, 7 and 8)**					
Being a girl	1.01(0.85–1.20)	1.07(0.85–1.35)	1.19(1.00–1.43)	1.13(0.89–1.44)	**1.40(1.22–1.62)**	**1.35(1.11–1.64)**
After-school LTPA (hour)	1.00 (1.00–1.00)	1.00 (1.00–1.00)	1.00 (1.00–1.00)	1.00(1.00–1.00)	1.00 (1.00–1.00)	1.00 (1.00–1.00)
Commuting time (hour)	1.00 (1.00–1.00)	1.00 (1.00–1.00)	1.00(0.99–1.00)	0.99(0.99–1.00)	1.00 (1.00–1.00)	1.00 (1.00–1.00)
Homework time (hour))	**1.10(1.02–1.19)**	1.05(0.95–1.17)	**1.22(1.12–1.32)**	**1.18(1.07–1.31)**	**1.25(1.17–1.38)**	**1.16(1.07–1.27)**
Screen time >= 2 hours	**1.41(1.08–1.84)**	1.15(0.80–1.67)	1.01(0.75–1.36)	0.79(0.52–1.21)	1.21(0.96–1.53)	1.06(0.77–1.46)
Playing mobile phone (hour)	**1.25(1.04–1.51)**	1.20(0.95–1.51)	**1.39(1.15–1.67)**	**1.27(1.00–1.61)**	**1.58(1.33–1.88)**	**1.37(1.10–1.71)**

^a^ All models were adjusted for age, BMI categories, parents’ education and whether or not lived with a sibling.

## Discussion

We investigated sleep duration, schedule and quality in a large sample of 4^th^ to 8^th^ grade school students living in metropolitan Shanghai. In our study, the average sleep duration was 9 hours and the time duration decreased with age. The result is similar to that reported in other Chinese studies of urban school-aged children [31–32] and Shanghai local government report. For example, in Shanghai, in the 2011 Green Standard Assessment of the Academic Performance (GSAAP) found that less than 50% of the Grade 4 students who slept more than 9 hours. [33] A recent meta-analysis has shown that Asian children have shorter weekday sleep duration compared with children in Australia, Europe and USA. [34] In contrast, we found that the sleep duration of the current sample was similar to counterparts in European countries [35–36] but longer than that of children from other Asian countries/regions such as Korea, [37] Malaysia [16] and Hong Kong. [38] Considering the different measures across studies, the comparisons among countries should be interpreted with caution. Within Shanghai, In 2011 GSAAP also found that there were less than 20% of the Grade 9 students who slept more than 8 hours. [33] Our study did not include Grade 9 students who face much more study load and academic pressure because of junior to high school graduation exam. In such case, it is possible our results may overestimate the sleep duration of Shanghai school-aged children in general.

A major finding of our study is that girls slept less and went to bed later compared with boys, and these differences increased with age. Although a sex difference in school night bedtime among urban Chinese children (5–12 years old) has been previously reported, [31] the age-sex interaction on sleep among Chinese children has not been reported. Our finding is similar to one Spanish study [39] which showed the difference of sleep duration between boys and girls varied with age among older adolescents. However, in other studies, the sex differences in sleep behaviors were either not significant [31] or in the opposite direction. [40] In our study, the most frequently reported sleep problem was daytime tiredness, ranging from one quarter to almost 40%, with the prevalence increasing with age and being higher among girls. Boys in elementary school (Grade 4 and 5) were at higher risk of having difficulty initiating sleep and maintaining sleep compared to girls of the same grade. The sex difference observed in this study suggests that boys and girls at each developmental stage are susceptible to different types of sleep problems.

We found consistent associations between secondary students’ time spent on homework and sleep duration and bedtime, after adjusting for perceived academic pressure. Academic performance has always been highly valued within Asian families and societies. In our study, homework time in secondary students was also associated with poorer sleep quality. Such findings highlight the potentially adverse effect of academic burden placed on Asian children which is a cultural phenomenon that is distinct from many Western populations.

More time spent on LTPA was associated with longer sleep duration and earlier bedtime among older girls. There have been similar findings among Japanese adolescents [41] for sleep duration and American adolescents [42] for sleep quality in cross-sectional studies. A possible explanation for the observed association among girls, but not among boys, was the beneficial effect of LTPA on sleep could be more pronounced among people who had more sleep complaints or disturbance. [43–44] In our sample, older girls reported more problems with sleep and the moderate association may be more easily detected in this subgroup.

Interestingly, school commuting time was inversely associated with sleep duration but not bedtime, a finding which may reflect the inability of school students to adjust their schedule to compensate for sleep loss caused by daily commuting to/from school. In Chinese cities, while parents are encouraged to send their children to local schools, many parents send their children to academically prestigious schools which may well be out of area, with subsequent longer commuting times. Our finding suggests there is a need to inform parents of the health benefits of both providing children with bedtime rules and strategies to address time issues associated with school commuting.

We examined the unique relationship between mobile phone playing and sleep. Mobile phone playing was inversely associated with sleep duration and bedtime; it was also associated with difficulty in maintaining sleep and daytime tiredness among adolescents in secondary school. One Canadian study found that access to mobile phones but not the duration of the usage was associated with shorter sleep duration among elementary students [45], while findings from Taiwan showed that such relationships could not be confirmed in adolescents. [46] In our study, mobile phone playing (i.e. surfing the internet and playing games), instead of total usage, was measured. Thus, the inverse association found in our study may reflect the negative influence of certain functions of mobile phone use on sleep. Future studies should measure each type of mobile phone usage based on the nature of the behavior.

In our sample, surveyed in 2011, 56.4% of children and adolescents owned personal mobile phones (results not shown). Given the exponential growth of smart phones that mimic functions that were once only available on desk-top computers, and recognizing that China has already become the leading consumer market for mobile phones, [47] we expect that short sleep duration and poor sleep quality attributed to mobile devices are likely to increase in the future. Information on types of phones (i.e., smart vs. traditional phones) and specific functions of mobile phone that children playing with could better inform the projection of mobile-phone related sleep loss of Chinese children.

It should be noted that spending ≥2 hours per day on screen-time for entertainment was not consistently associated with our measures of sleep quality. A separate study conducted among the Chinese pediatric population, six years prior to our study, found that more than 2 hours’ TV viewing time was linked to later bed time, but not sleep duration, among elementary students. [23] A possible explanation could be that, on school days, TV viewing and playing computers are more likely to be limited within the family, especially among primary school students, while secondary students tend to use mobile phones for communication in the evening, possibly resulting in interrupted sleep and daytime tiredness. [48] Thus, a practical solution would be to keep all electronic devices out of children’s bedrooms and to restrict night use of mobile phones, especially after children go to bed.

### Strengths and limitations

To our knowledge, this is the first study to investigate the association between after-school activities and sleep among a Chinese child and adolescent population. Study strengths include the large representative sample, the high response rate, and comprehensive measures of sleep outcomes. Although questions of sleep problems are indicators, not clinical measures, of sleep quality, the findings provide useful population information of sleep hygiene in Chinese school-aged children. The inclusion of mobile phone playing was a novel aspect of this study and shows that mobile screen technologies may play a critical role in children’s sleep hygiene, and hence there is a need to monitor children’s engagement with these technologies.

Limitations to take into account when interpreting our results include self-report data, which may be subject to recall bias; however, a separate study shown good agreement and correlation between self-report and objective measures of sleep especially on school nights. [49] Other factors, such as bed sharing and parents’ sleep habits, were not included in the study, nor did we have measures of sleep during non-school days. We are aware that sleep behaviors and outcomes and relevant risk factors could differ between school and non-school days.

## Conclusion

Girls slept less and reported more sleep problems. Mobile phone playing had a small but significant association with sleep duration, schedule, and quality in secondary school students. The findings build evidence for formulating intervention strategies to change unhealthy practices associated with poor sleep hygiene, especially among adolescents. Simple measures such as setting a bed time and restricting children’s use of mobile phones after school are implicated. However, a remaining challenge is how to address the academic pressure felt by Chinese children, given the cultural emphasis the Chinese population places on education.
